# Antibody cross-linking and target elution protocols used for immunoprecipitation significantly modulate signal-to noise ratio in downstream 2D-PAGE analysis

**DOI:** 10.1186/1477-5956-9-45

**Published:** 2011-08-04

**Authors:** Mirta ML Sousa, Kristian W Steen, Lars Hagen, Geir Slupphaug

**Affiliations:** 1 Department of Cancer Research and Molecular Medicine and The FUGE proteomics laboratory, Norwegian University of Science and Technology, N-7006 Trondheim, Norway

## Abstract

**Background:**

Immunoprecipitation and subsequent 2D-PAGE/mass spectrometry are powerful tools to study post-translational protein modifications. Often disregarded in this workflow is the impact of the chemical cross-linker upon antibody affinity, as well as incomplete elution of primary target protein in buffers commonly used in 2D-PAGE. This may impede detection of non-abundant protein isoforms.

**Results:**

Here we have compared cross-linking of antibodies to Dynabeads^® ^Protein A by using DMP or BS^3^, as well as the efficiency of various target elution buffers prior to 2D-PAGE separation. BS^3 ^cross-linking generally resulted in less non-specific binding than DMP, whereas DMP cross-linking gave overall higher yield of target protein. Regardless of the cross-linker used, incomplete elution of target protein was observed with conventional glycine- or urea-based buffers. Conversely, complete elution was obtained with 2% hot SDS and subsequent dilution in urea buffer containing 4% CHAPS, to 0.2% final SDS yielded perfectly focused gels suitable for mass spectrometry analysis.

**Conclusion:**

Careful choice of Ig cross-linker as well as efficient elution of target protein in SDS prior to downstream 2D-PAGE may be key factors to analyze low-abundance proteins enriched by magnetic bead immunoprecipitation.

## Background

Immunoprecipitation (IP), particularly in the magnetic bead format, is a highly efficient method to enrich endogenous proteins from complex mixtures [1]. It is especially convenient for analysis of low abundance proteins and protein isoforms, and is by far faster than conventional column chromatography methods that include the additional risk of introducing artificial protein modifications due to the often lengthy purification schemes. To investigate potential isoforms of a given protein, 2D-PAGE [2] is very informative since it allows exquisite separation in two dimensions and a readable output as image. Thus potential isoforms resulting from e.g. alternative splicing, heterozygous polymorphisms as well as many post-translational modifications can often be visually observed (recently reviewed in [3-5]). Such modifications can then be further analyzed and sometimes quantified by appropriate mass spectrometry techniques [6].

A general problem with IP, notwithstanding the downstream electrophoretic method, is that a large number of proteins other than the antigen are generally observed in the resultant gels and of which most are non-specifically bound to the affinity matrix. This is especially problematic in co-immunoprecipitation experiments aiming at identification of protein:protein interactions. Here, the IP conditions are often adjusted to increase signal to noise-ratio (short incubation times, low temperature) and IP- and wash buffers chosen that maintain the integrity of protein complexes [7]. When the primary aim is detailed study of a non-abundant protein or isoforms thereof, present at often minute amounts in the cell, other considerations may be more important. Increased incubation times may be employed to ensure maximum binding of the target protein, while stringent washes can be applied to reduce non-specific background without interrupting antibody:target binding. To avoid contaminating immunoglobulins in the final eluate, antibodies are often covalently cross-linked to the matrix. Efficient reagents for cross-linking of antibodies can to a large extent eliminate Ig leakage and thus allow re-usage of the beads subsequent to elution. However, cross-linking may also modulate the efficiency of antigen binding [8]. Cross-linking to protein A- or protein G coated matrices [9] is commonly used, since immunoglobulins bind these proteins via their Fc regions, and thus leave the variable regions accessible for antigen binding. The most common method until now to covalently link IgGs to magnetic protein A or G beads has been dimethyl pimelimidate (DMP) [10]. DMP is a diimido ester that reacts with primary α- and ε-amines in proteins, with a preference for ε-amines of lysines at pH 9-10 [11]. Recently, DMP has been replaced by bis[sulfosuccinimidyl] suberate (BS^3^) in the protocols from several protein A/G bead vendors including Dynabeads^®^. BS^3 ^is an *N*-hydroxysulfosuccinimide (NHS) ester that also targets the primary amine groups, but have additional cross-reactivity towards other nucleophilic groups in proteins, including tyrosines, serines and threonines [12,13].

In most protocols involving IP, proteins are eluted from the beads prior to downstream analyses. Surprisingly, this important step has received relatively little attention. When preserving biological activity of proteins is not an issue, such as in protocols involving one-dimensional PAGE (1DE) and western analysis, it is common to elute target proteins directly into hot SDS or LDS-containing loading buffers. This harsh elution essentially strips the beads of bound proteins, but also renders the beads unusable for repeated use. It is thus not uncommon to elute target proteins in e.g. glycine-HCl at pH 2.5-2.8, that generally (but not always) maintains antibody integrity[14], anticipating that this buffer elutes target protein as efficient as LDS or SDS. If IP is followed by two-dimensional-PAGE (2DE), other elution procedures are also regularly employed, commonly containing the constituents of isoelectric focusing (IEF) buffers. IEF buffers are optimized to maintain protein solubility, and generally contain urea, thiourea, a non-ionic detergent such as NP-40 or a zwitterionic detergent such as CHAPS and a reducing agent such as DTT. Moreover, it is important to keep the concentration of salt low, since elevated conductivity and electroendoosmosis impairs focusing of the proteins[15]. Charged detergents such as SDS are also generally avoided since they may compromise the IEF step[16]. To separate proteins isolated by IP in 2DE, it is instead common to elute the proteins into buffers that contribute little to the conductivity in the IEF step, such as urea-CHAPS-buffer or the ready made DeStreak™ solution (GE Healthcare). This also circumvents cumbersome pre-IEF cleanup steps such as precipitation or dialysis with the additional risk of losing proteins present in small quantities. A potential drawback of such elution methods and that is not realized by many researchers, is that the elution efficiency of the target protein may not be as efficient as the hot SDS-elution generally employed for 1DE analysis. Thus results from downstream 2DE, 2D-western and mass spectrometry analysis may potentially lead to erroneous conclusions.

In this study we have addressed the effect of the two commonly used cross-linkers DMP and BS^3 ^to couple antibodies to paramagnetic protein A- or protein G-coated beads, upon the degree of non-specific protein binding to Dynabeads^® ^protein A during IP. We moreover compared different protocols used to elute target proteins from the beads and demonstrate that buffers commonly employed prior to 2DE result in variable and often poor elution. Conversely, heating of the beads in a buffer containing 2% SDS was the most effective overall method. Importantly, target proteins eluted from the Dynabeads^® ^could be successfully separated in 2DE by diluting the SDS in urea buffer containing 4% CHAPS without compromising the IEF separation. The increased amount of target protein recovered from beads may be key to allow identification and analysis of low-abundant proteins and protein isoforms enriched by immunoprecipitation.

## Results and Discussion

### Analysis of DMP and BS^3 ^cross-linkers

**Figure 1:**
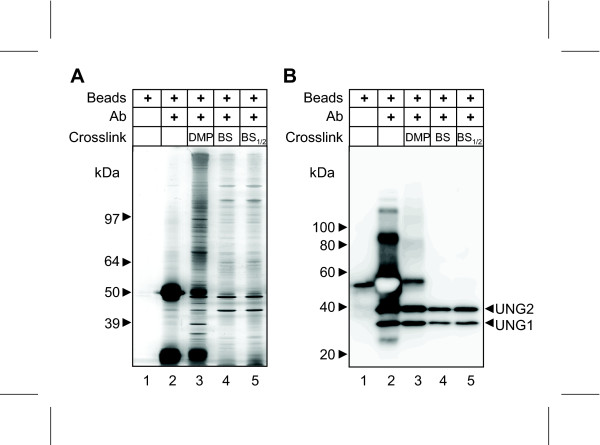
**Type of cross-linker affects specificity and yield of immunoprecipitated protein**. Dynabeads Protein A were bound to anti-UNG PU59 antibodies (Ab) and either incubated directly with HeLa cell extract, or cross-linked with DMP, BS^3 ^(BS), or half the recommended amount of BS^3 ^(BS_1/2_) prior to incubation with HeLa cell extract for 4 hours. Subsequent to washing, bound proteins were eluted in SDS-PAGE loading buffer and analyzed by: A) Silver stained gel; B) Western blot. The bright region in lane 2 of the western blot is caused by excessive binding of HRP-conjugated antibody to the primary antibody eluting from the non-crosslinked beads, causing exhaustion of the luminol substrate.

We first addressed the degree of non-specific protein binding to Dynabeads^® ^protein A. These paramagnetic beads were chosen over Sepharose or agarose beads due to their relative ease of handling, minimal loss of protein and the negligible degree of buffer carry-over between washing steps subsequent to IP. Moreover, magnetic Dynabeads^® ^were recently shown to have an overall lower non-specific binding of nuclear proteins, our primary focus of interest, than Sepharose and agarose beads [17]. Firstly, "naked" Dynabeads^® ^protein A were washed in PBS-T and incubated directly with Hela cell extract. Subsequent to washing of the beads and complete elution in hot LDS very few bands were observed in the silver stained gel (Figure 1A, lane 1). In the western blot, one band was observed at about 50 kDa, likely representing a small degree of leakage of protein A (Figure 1B, lane 1). Thus, under the present experimental conditions, the protein A beads do not contribute significantly to non-specific protein binding. Next, beads were incubated with anti-UNG PU59 antibodies and left either non-covalently attached or cross-linked using DMP or BS^3^. PU59 targets the common catalytic domain of mitochondrial UNG1 and nuclear UNG2, which migrate at about 33 kDa and 40 kDa, respectively, in SDS-PAGE. The UNG proteins are not abundant in the cells and are barely visible in silver-stained gels (Figure 1A) subsequent to IP under the present conditions. It is thus not likely that any specific interaction partner of the UNG proteins should constitute distinct visible bands in the gel. Consequently, most of the other bands subsequent to IP likely represent non-specifically bound proteins. The antibody-coated beads displayed a marked increase in overall protein binding compared to beads alone (Figure 1A, lane 2). As expected, in the absence of cross-linker, massive amounts of IgG_H _and IgG_L _co-eluted from the beads in hot LDS, and obstructed significant parts of the western blot. Nevertheless, strong bands were observed for UNG1 and UNG2 (Figure 1B, lane 2). Cross-linking using DMP removed most, but not all Ig leakage without compromising the UNG western signals. Moreover, protein A leakage was completely eliminated (Figure 1B, lane 3). However, DMP also resulted in a marked increase in non-specifically bound proteins (Figure 1A, lane 3). By comparison, no trace of Ig (or protein A) leakage was observed after BS^3 ^cross-linking, and this also resulted in significantly lower levels of non-specifically bound protein than with DMP (Figure 1A,B, lane 4). Notably, cross-linking using BS^3 ^also reduced the UNG signals in the western blot. Nevertheless, an excellent western signal-to-noise ratio was obtained by cross-linking the antibodies using BS^3 ^prior to IP, and this was maintained even if the amount of BS^3 ^in the reaction was reduced to half of that recommended by the bead manufacturer (BS_1/2_, Figure 1A,B, lane 5). Since the considerably higher cost of BS^3 ^compared to DMP (~30 × higher per coupling reaction) may be a concern in many laboratories, the reduced concentration of BS^3 ^was maintained in the following experiments to monitor elution efficiency of target proteins.

### Efficiency of target protein elution in varying buffers

**Figure 2:**
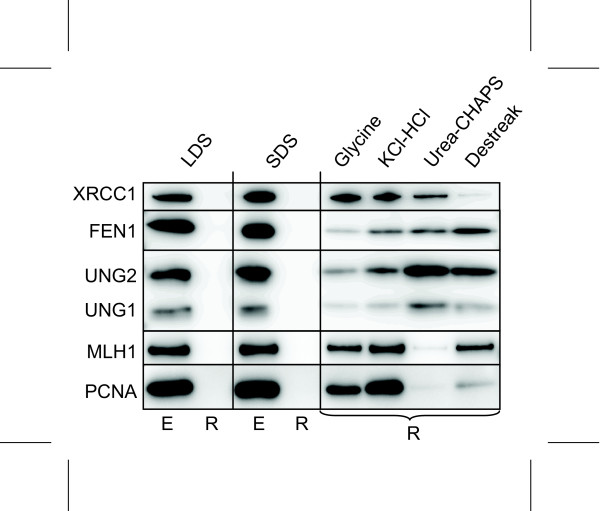
**Elution conditions differentially affect elution from different antibodies**. Comparison of commonly used protocols to elute target proteins from Dynabeads^® ^Protein A and other bead-based immunomatrices. Elution in hot LDS or SDS buffers generally employed in 1DE protocols results in complete removal of bound proteins (E; eluate, R; residual on beads as monitored by a second round of hot LDS/SDS treatment). In contrast, elution in four commonly used buffers used for downstream 2DE-analysis consistently results in considerable residual target protein remaining on the beads (only residual protein on beads are shown, as monitored by a second elution in hot LDS buffer). Note that the amount of residual protein on the beads is affected both by the elution conditions as well as the type of antibody.

To monitor the efficiency of commonly used elution buffers to release target proteins, Protein A beads were cross-linked to antibodies against XRCC1, FEN1, UNG1/2, MLH1 or PCNA, and incubated with HeLa cell extracts as above. Subsequent to washing, target proteins were eluted in LDS buffer, SDS buffer, glycine (pH 2.5), KCl-HCl (pH 1.5), urea-CHAPS buffer or Destreak rehydration solution (GE Healthcare). As expected, elution in hot LDS or SDS effectively stripped the target proteins off the beads, and no western signals were obtained for these proteins after a second round of elution (Figure 2 E; first eluate in hot LDS or SDS, R; residual protein on beads as monitored from a second round of LDS elution). In contrast, elution in any of the other buffers yielded considerable amounts of target protein left on the beads (Figure 2). Moreover, the performance of the four buffers was highly antibody-specific, likely reflecting the type of bonds dominating the antibody-antigen interaction. Whereas the acidic glycine and KCL-HCL buffers eluted most of the FEN1 and UNG proteins, large amounts of XRCC1, MLH1 and PCNA remained on the beads. Conversely, urea-CHAPS effectively eluted MLH1 and PCNA whereas this buffer performed poorly with XRCC1 and UNG1/2. Strikingly, the Destreak solution, containing thiourea in addition to urea-CHAPS eluted XRCC1 much more effectively than the urea-CHAPS, whereas the opposite was found with MLH1 (Figure 2). These results strongly indicate that buffers commonly used to elute antigens for downstream 2DE result in variable and often poor elution, unless thorough testing of elution conditions is undertaken for each antibody:protein pair.

**Figure 3:**
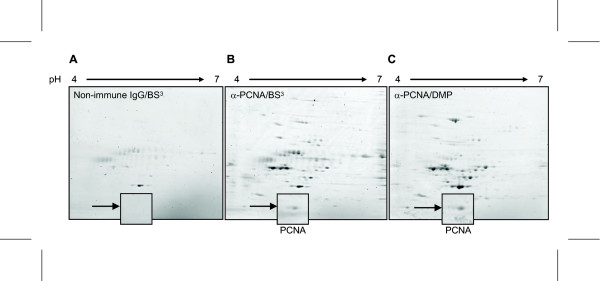
**SDS elution from Dynabeads^® ^protein A with 2% SDS is fully compatible with downstream 2DE**. Eluted HeLa cell proteins from beads cross-linked to non-immune mouse IgG (A), anti-PCNA using BS^3 ^as cross-linker (B), or anti-PCNA using DMP as cross-linker (C). Samples were used to rehydrate 7 cm IPG strips, pH 4-7 overnight. Proteins were stained using Sypro Ruby. PCNA spots are indicated by arrows.

To circumvent such laborious testing, we next aimed to combine the highly efficient elution by hot SDS with downstream 2DE without compromising the 1^st ^dimension IEF step. The use of SDS in sample preparations prior to IEF was described more than a decade ago, as a means to ensure solubility of yeast proteins [18]. To implement SDS elution in the Dynabeads^® ^protein A IP/2DE protocol, PCNA was immunoprecipitated using a monoclonal anti-PCNA antibody cross-linked to Protein A beads using either DMP or BS^3^. Beads cross-linked to a none-immune antibody using BS^3 ^were included as controls to monitor non-specific binding. PCNA gives a somewhat higher yield subsequent to immunoprecipitation than the other proteins illustrated in Figure 2, and was thus targeted to allow a visual evaluation of the signal-to-noise ratio subsequent to Sypro Ruby-staining of the resultant 2D-PAGE gels (Figure 3). Notably, all three gels are nicely focused, demonstrating the compatibility of SDS elution with downstream isoelectric focusing when either BS^3 ^or DMP cross-linked antibodies are employed. In agreement with the results illustrated in Figure 1, BS^3 ^cross-linking resulted in a somewhat different profile of non-specific binding proteins than DMP (Figure 3B,C). More importantly, however, the level of target PCNA obtained by DMP-cross-linking was repeatedly higher than that obtained with BS^3^-cross-linking, resulting in unequivocal identification of PCNA by MALDI-TOF/TOF analysis only in the DMP sample (data not shown). Thus, the choice of cross-linker may be crucial to enrich sufficient amount of target for downstream mass spectrometry analysis.

**Figure 4:**
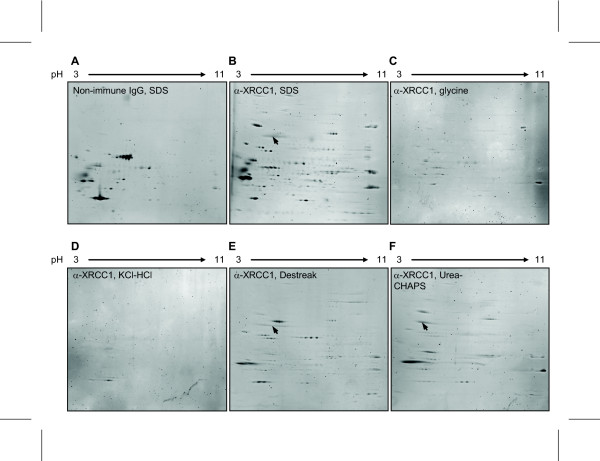
**Choice of elution buffer is crucial for identification of non-abundant proteins**. 2DE protein patterns after IP performed with beads cross-linked to anti-normal rabbit IgG (A) or anti-XRCC1 antibody (B-F) using BS^3^. Bound proteins were eluted using SDS (A,B), glycine (C), KCl-HCl (D), Destreak (E) and urea-CHAPS (F) buffers. Proteins were stained using Sypro Ruby.

Next we compared the different elution buffers illustrated in Figure 1, prior to downstream 2DE and using XRCC1 as target. XRCC1 is a non-abundant protein existing as multiple post-translationally modified forms in the cell, and may interact with a multitude of proteins involved in DNA repair [19]. Both the presence in larger protein complexes as well as potential modifications constitutes challenges for efficient capture by antibodies. Thus we employed a polyclonal anti-XRCC1 antibody to allow binding to multiple epitopes, and cross-linking with BS^3 ^to minimize non-specific binding. IP using non-immune rabbit IgG was included as control. Eluted proteins were separated in IPG 3-11 strips, and the resulting 2DE gels (Sypro Ruby stained) are illustrated in Figure 4. Again, elution in SDS yielded excellently focused gels (Figure 4A,B). Native XRCC1 has a molecular mass of 69.5 kDa, and a calculated pI of 6.02. Excision of spots in the gel area corresponding to the expected migration of XRCC1, and subsequent MALDI-TOF/TOF analysis (table 1) resulted in unequivocal identification of XRCC1 in the SDS-eluted sample (arrow, Figure 4B). Interestingly, the protein profiles of the 2DE gels obtained with the different elution buffers are very different. Very few proteins could be observed subsequent to elution in the acidic glycine- and KCl-HCL buffers (Figure 4C,D), and none in the expected area of XRCC1. These results are in agreement with the considerable amount of target XRCC1 remaining on the protein A beads subsequent to elution in these buffers (Figure 2). Conversely, XRCC1 was clearly identified subsequent to elution in Destreak and urea-CHAPS buffers (Figures 4E,F) and sufficient to allow identification by MALDI-TOF/TOF (table 1). This is in agreement with the low level of residual XRCC1 on the beads subsequent to elution in these buffers (Figure 2).

**Table 1:** XRCC1 was identified by MALDI peptide mass fingerprinting subsequent to elution from Protein A beads with SDS, Urea-CHAPS or Destreak solution.

Elution buffer	Mascot score	Number of peptides
SDS	205	15
Urea-CHAPS	118	10
Destreak	67	7

SDS elution yielded significantly better score value and higher number of identified peptides than the other two elution buffers.

When employing SDS buffer to elute target proteins it is important that a) the final concentration of SDS in the sample loaded onto IEF strips does not exceed 0.2% and b) a sufficient volume of SDS buffer is used to cover the protein A beads and to allow complete elution. The volume of protein A beads (measured as original bead suspension) used in IP should be 2.5-3 × that of the maximum allowed SDS elution buffer in the IEF step. Thus, for small (7 cm) IEF strips accommodating up to 130 μL final sample, we have used 13 μL 2% SDS buffer to completely elute bound target protein from 40 μL Dynabeads protein A. Correspondingly, for 24 cm IEF strips that can accommodate 450 μL samples we have used 45 μL 2% SDS buffer to completely elute proteins from 120 μL Dynabeads protein A. Notably, however, even larger volumes of beads can be employed by dividing the washed beads into two or more tubes, and performing successive elution in each of the tubes into the same volume of SDS buffer. We routinely achieve complete elution from 200 μL Dynabeads protein A in 45 μL SDS elution buffer by dividing the beads into 2 × 100 μL batches and performing successive elution. After eluting with SDS buffer, we routinely wash the beads with urea buffer containing 4% CHAPS, to ensure full recovery of the eluted proteins.

## Conclusions

In the present work we have compared the commonly used cross-linkers BS^3 ^and DMP in the coupling of antibodies to magnetic protein A Dynabeads. We find that a main advantage of BS^3 ^is general lower non-specific binding of proteins compared to DMP. However, BS^3 ^also results in an overall lower binding of some target proteins, likely reflecting its propensity to react with tyrosines, serines and threonines present in the epitope binding regions of immunoglobulins [12,13]. Thus, for use in downstream 2DE protocols DMP may be the preferred alternative, especially for targeting basic proteins that generally display somewhat lower non-specific binding than acidic proteins (Figure 4). In addition, the considerably lower cost of DMP may be a concern for some laboratories.

We next tested different protocols to elute target proteins from Dynabeads protein A, and demonstrate that four buffers commonly used for elution prior to 2DE separation result in variable and often poor elution of the target proteins. In contrast, elution in 2% SDS buffer and subsequent washing in urea buffer containing 4% CHAPS resulted in complete elution of all the tested target proteins. Furthermore, dilution of the eluted material in urea-CHAPS buffer to a final concentration of 0.2% SDS was fully compatible with downstream 2DE separation and mass spectrometry analysis. Thus we strongly recommend the use of SDS to elute immunoprecipitated proteins from Dynabeads protein A prior to improve detection low-abundance proteins in 2DE and further analysis by mass spectrometry.

## Materials and methods

### Cell culture and preparation of lysates

Hela S3 cells were cultured in Dulbecco's Modified Eagle Medium supplemented with 10% (v/v) fetal bovine serum, 2 mM L-glutamine, 0.1 mg/mL gentamicin and, 2.5 μg/mL amphotericin B. Freely cycling HeLa S3 cells were harvested by trypsination and all subsequent steps were performed at 4°C. After 3x wash in PBS, cells were resuspended in one packed cell volume (PCV) buffer 1 (10 mM Tris-HCl pH 8.0, 200 mM KCl). Then 2 × PCV buffer 2 [10 mM Tris-HCl pH 8.0, 200 mM KCl, 2 mM EDTA, 40% glycerol, 0.5% NP40, 1 mM DTT, 1% phosphatase inhibitor cocktails 1 and 2 (Sigma), and 1.2 mg/mL Complete™ protease inhibitors (Roche)] was added and the cells were incubated by constant rotation for 1.5 h at 4°C. After clearing the lysates at 15,000 × g for 10 min, supernatants were collected and used for subsequent experiments.

### IgG cross-linking, immunoprecipitation and elution of target proteins

The following antibodies were cross-linked to Dynabeads^® ^Protein A (Dynal, Norway): Anti-UNG (PU59, in-house prepared rabbit polyclonal antibody against the common catalytic domain of UNG 1 and 2), anti-FEN1 (BL587, Bethyl laboratories) and anti-XRCC1 (SC-11429), anti-PCNA (SC-56), anti-MLH1 (SC-56159), non-immune mouse IgG (SC-2025) from Santa Cruz and in-house prepared non-immune rabbit IgG. Antibodies were covalently coupled to the beads using either DMP (Sigma) or BS^3 ^(Thermo Scientific) as cross-linkers according to the manufactures instructions (Dynal). IP with antibodies non-covalently bound to beads was performed by incubating the beads with antibodies for 1 hour prior to washing and incubation with cell extract. For direct comparison of different elution buffers, 120 μl of Dynabeads^® ^were incubated with HeLa cell extract (300 μg protein) under gentle rotation at 4°C for 4 h. Beads were then washed 3 times with 10 mM Tris-HCl pH 7.5, 50 mM KCl and equally split into 6 new tubes that were processed using different protein elution protocols. Elution in LDS or SDS was performed by heating the beads for 10 minutes at 70°C in 20 μl of NuPage loading buffer (Invitrogen) containing 100 mM DTT, or SDS elution buffer (2% SDS, 100 mM Tris-HCl pH 7.5, 10% glycerol, 0.5 mM EDTA, 100 mM DTT and bromophenol blue as tracer), respectively. Elution with glycine was performed by adding 20 μl of 50 mM glycine pH 2.5 to the beads, vortexing briefly and incubating with gentle rocking for 5 minutes. After removal of the supernatant, the procedure was repeated and the combined supernatants neutralized with 1 M Tris pH 10 (2 μL). Elution using KCl-HCl was performed similarly to that of glycine by using 0.2 M KCl-HCl pH 1.5. Elution with destreak or urea-CHAPS buffer (8 M urea, 4% CHAPS, 130 mM DTT) was performed by resuspending beads in 20 μL of either buffer and followed by incubation overnight at 4°C with constant agitation. Subsequent to the elution in any of the six buffers above, beads were heated for 10 minutes at 70°C in LDS buffer and these eluates were subjected to western analysis to monitor the amount of residual target protein remaining bound to the beads after the first elution.

To compare the 2D electrophoretic patterns obtained with the different elution methods, IPs were performed by incubating 80 μL of beads coupled to anti-XRCC1 antibody using BS^3 ^with 2 mg Hela cell extract overnight at 4°C. Beads were washed 3 times with 10 mM Tris-HCl, 50 mM KCl. For elution with SDS, and to minimize the final SDS concentration, samples were divided in 2 tubes containing 40 μL beads each. Sample 1 was then eluted in 13 μL SDS elution buffer for 10 minutes at 70°C. The eluate was then transferred to sample 2, and the second elution performed as above. To collect residual proteins, the two samples were washed sequentially with 117 μL urea-CHAPS buffer and combined with the SDS eluate to a final volume of 130 μL. Elution in glycine or HCl-KCl was performed as described above for 1DE, except that 2 × 40 μL of either buffer was used. The sample eluted with glycine was then reduced *in vacuo *to 15 μL and then added destreak solution to a final volume of 130 μL. The sample eluted with KCl-HCl was desalted in a spin column (Millipore) prior to dilution in Destreak solution to 130 μL. Elution with destreak or urea-CHAPS buffer were performed as described above, in 130 μL of either buffer. Finally, 1% IPGbuffer pH 3-11 (GE Healthcare) was added to each sample prior to rehydration into 7 cm IPG strips, pH 3-11 overnight. IEF was carried out according to manufacture's instructions (GE Healthcare).

### Electrophoresis

Eluted proteins were subjected to 1DE separation in 4-12% Novex Bis-Tris gels using MOPS running buffer (Invitrogen) prior to silver staining or western blot analysis. In 2DE, isoelectric focusing was conducted in Ettan IPGphor II (GE healthcare). After IEF, strips were incubated in equilibrium buffer (50 mM Tris-HCl pH 8.8, 6 M urea, 30% glycerol, 2% SDS) containing 1% DTT for 15 minutes, then in the same buffer containing 2.5% iodoacetamide instead of DTT for 15 minutes. Second dimension SDS-PAGE was performed using 4-12% Novex Bis-Tris IPGwell gels (Invitrogen). Gels were stained by Sypro Ruby protein stain according to the manufacturers instructions (Molecular Probes, Invitrogen) and scanned using a Typhoon Trio variable mode imager™ (GE Healthcare).

#### Western blot analysis

Proteins separated by 1DE or 2DE were transferred to PVDF-membranes (Immobilon, Millipore). The membranes were blocked for 1 h in 5% fat-free dry milk in PBS containing 0.1% Tween and then incubated for 1 h in primary antibody diluted in blocking buffer. After washing 3 × 10 min in PBS-T, membranes were incubated with HRP-conjugated swine anti-rabbit (P0399) or HRP-conjugated rabbit anti-mouse (P0260) secondary antibodies (Dako Denmark) in blocking buffer for 1 h. After 4 × 10 min washes in PBS-T blots were developed using SuperSignal West Femto Maximum Sensitivity Substrate (Thermo Scientific) and scanned in a Kodak IS4000R imager (Fisher Scientific).

### Mass spectrometry

Manually excised spots were subjected to in-gel trypsination (Promega) as described [20]. Peptides were desalted as described [21] and mixed with α-cyano-4-hydroxycinnamic acid (CHCA) prior to MALD-TOF/TOF mass spectrometry (Ultraflex III, Bruker). MS and MS/MS data were used in subsequent searches by Mascot software version 2.2 (http://www.matrixscience.com/, Matrix Science) using the MSDB protein sequence database for human proteins.

## List of abbreviations

CHAPS: 3-[(3-cholamidopropyl)dimethylammonio]-1-propanesulfonate; FEN1: Flap structure-specific endonuclease 1; LDS: lithium dodecyl sulphate; MALDI-TOF: matrix-assisted laser desorption/ionization-time-of-flight; MLH1: MutL homolog 1; NP-40: nonyl phenoxypolyethoxylethanol; PBS-T: phosphate buffered saline, 0,1% Tween 20; PAGE: polyacrylamide gel electrophoresis; PCNA: proliferating cell nuclear antigen; SDS: sodium dodecyl sulphate; UNG: Uracil-DNA glycosylase encoded by the *UNG *gene; XRCC1: X-ray repair complementing defective repair in Chinese hamster cells 1.

## Competing interests

The authors declare that they have no competing interests.

## Authors' contributions

MMLS and KWS contributed equally to this study. Both conceived of the study, carried out immunoprecipitations, eletrophoretic separations and interpreted the data. LH maintained cell cultures and prepared the cell extracts. MMLS conducted the MS analyses. GS contributed to the design of the study, interpretation of data and helped draft the manuscript. All authors read and approved the final manuscript.

## References

[B1] Kaboord B Perr M Isolation of proteins and protein complexes by immunoprecipitation Methods Mol Biol 2008 424 349 64 10.1007/978-1-60327-064-9_27 18369874

[B2] O'Farrell PH High resolution two-dimensional electrophoresis of proteins J Biol Chem 1975 250 4007 21 236308 PMC2874754

[B3] Chevalier F Highlights on the capacities of "Gel-based" proteomics Proteome Sci 2010 8 23 10.1186/1477-5956-8-23 20426826 PMC2873371

[B4] Gorg A Drews O Luck C Weiland F Weiss W 2-DE with IPGs Electrophoresis 2009 30 Suppl 1 S122 32 19441019 10.1002/elps.200900051

[B5] Issaq H Veenstra T Two-dimensional polyacrylamide gel electrophoresis (2D-PAGE): advances and perspectives Biotechniques 2008 44 697 8 700 10.2144/000112823 18474047

[B6] Hagen L Kavli B Sousa MM Torseth K Liabakk NB Sundheim O Pena-Diaz J Otterlei M Horning O Jensen ON Cell cycle-specific UNG2 phosphorylations regulate protein turnover, activity and association with RPA EMBO J 2008 27 51 61 10.1038/sj.emboj.7601958 18079698 PMC2147998

[B7] Ten Have S Boulon S Ahmad Y Lamond AI Mass spectrometry-based immuno-precipitation proteomics - The user's guide Proteomics 2011 11 1153 9 10.1002/pmic.201000548 21365760 PMC3708439

[B8] Cress MC Ngo TT Site specific immobilization of immunoglobulins Am Biotech Lab 1989 7 16 9

[B9] Gersten DM Marchalonis JJ A rapid, novel method for the solid-phase derivatization of IgG antibodies for immune-affinity chromatography J Immunol Methods 1978 24 305 9 10.1016/0022-1759(78)90133-3 82592

[B10] Schneider C Newman RA Sutherland DR Asser U Greaves MF A one-step purification of membrane proteins using a high efficiency immunomatrix J Biol Chem 1982 257 10766 9 6955305

[B11] Hunter MJ Ludwig ML The reaction of imidoesters with proteins and related small molecules J Am Chem Soc 1962 84 3491 504 10.1021/ja00877a016

[B12] Kalkhof S Sinz A Chances and pitfalls of chemical cross-linking with amine-reactive N-hydroxysuccinimide esters Anal Bioanal Chem 2008 392 305 12 10.1007/s00216-008-2231-5 18724398

[B13] Madler S Gschwind S Zenobi R Role of arginine in chemical cross-linking with N-hydroxysuccinimide esters Anal Biochem 2010 398 123 5 10.1016/j.ab.2009.11.020 19931213

[B14] Firer MA Efficient elution of functional proteins in affinity chromatography J Biochem Biophys Methods 2001 49 433 42 10.1016/S0165-022X(01)00211-1 11694292

[B15] Rabilloud T Vaezzadeh AR Potier N Lelong C Leize-Wagner E Chevallet M Power and limitations of electrophoretic separations in proteomics strategies Mass Spectrom Rev 2009 28 816 43 10.1002/mas.20204 19072760

[B16] Zuobi-Hasona K Crowley PJ Hasona A Bleiweis AS Brady LJ Solubilization of cellular membrane proteins from Streptococcus mutans for two-dimensional gel electrophoresis Electrophoresis 2005 26 1200 5 10.1002/elps.200410349 15706571

[B17] Trinkle-Mulcahy L Boulon S Lam YW Urcia R Boisvert FM Vandermoere F Morrice NA Swift S Rothbauer U Leonhardt H Lamond A Identifying specific protein interaction partners using quantitative mass spectrometry and bead proteomes J Cell Biol 2008 183 223 39 10.1083/jcb.200805092 18936248 PMC2568020

[B18] Harder A Wildgruber R Nawrocki A Fey SJ Larsen PM Gorg A Comparison of yeast cell protein solubilization procedures for two-dimensional electrophoresis Electrophoresis 1999 20 826 9 10.1002/(SICI)1522-2683(19990101)20:4/5<826::AID-ELPS826>3.0.CO;2-A 10344254

[B19] Almeida KH Sobol RW A unified view of base excision repair: lesion-dependent protein complexes regulated by post-translational modification DNA Repair (Amst) 2007 6 695 711 10.1016/j.dnarep.2007.01.009 PMC1995033 17337257

[B20] Shevchenko A Wilm M Vorm O Mann M Mass spectrometric sequencing of proteins silver-stained polyacrylamide gels Anal Chem 1996 68 850 8 10.1021/ac950914h 8779443

[B21] Rappsilber J Ishihama Y Mann M Stop and go extraction tips for matrix-assisted laser desorption/ionization, nanoelectrospray, and LC/MS sample pretreatment in proteomics Anal Chem 2003 75 663 70 10.1021/ac026117i 12585499

